# 201. Large Scale Implementation of Opportunistic HCV Treatment during Hospitalization in a US Tertiary Care Hospital

**DOI:** 10.1093/ofid/ofaf695.073

**Published:** 2026-01-11

**Authors:** L Madeline McCrary, Megan R Curtis, Zoe R Leyh, Patricia Werner, Jessica R Elrod-Gallegos, Patrick Kojima, Lauren Karpman, Jami Cain, Katy Vora, Michael J Durkin, Tracey Habrock-Bach, Laura R Marks

**Affiliations:** Washington University School of Medicine, St. Louis, MO; Washington University School of Medicine, St. Louis, MO; Washington University in St. Louis, SAINT LOUIS, Missouri; Washington University School of Medicine, St. Louis, MO; Washington University School of Medicine, St. Louis, MO; Washington University in St. Louis, SAINT LOUIS, Missouri; Washington University in St. Louis, SAINT LOUIS, Missouri; Washington University in St. Louis, SAINT LOUIS, Missouri; Washington University in St. Louis, SAINT LOUIS, Missouri; Washington University School of Medicine, St. Louis, MO; Washington University School of Medicine, St. Louis, MO; Washington University in St. Louis, SAINT LOUIS, Missouri

## Abstract

**Background:**

Hepatitis C virus (HCV) remains a leading cause of liver-related morbidity and mortality. Despite effective treatment with direct-acting antivirals (DAAs), many individuals remain untreated due to systemic barriers. Hospitalization offers a “reachable moment” to initiate HCV care, particularly for underserved populations. We present the preliminary findings from a navigator-supported opportunistic HCV treatment protocol initiated during admission to a US tertiary care hospital.

**Methods:**

Navigators included a case manager and nurse navigator who assisted with review of inpatients with detectable HCV RNA, provided education, verified insurance coverage, and referred eligible patients for infectious diseases consultation. Consultations were billed to primary payors. DAAs were prescribed during hospitalization via outpatient specialty pharmacies. Medications were delivered bedside at the time of discharge when feasible. Priority populations included individuals with transportation barriers, prior missed outpatient appointments, discharges to supervised settings, people who use drugs, and postpartum women.

**Results:**

174 people initiated DAA therapy through the opportunistic treatment protocol. To date, 136 patients were due for sustained virologic response (SVR) labs among whom 76% were known to have achieved SVR and/or treatment completion. The remaining individuals are still completing treatment or not yet due for SVR labs.

**Conclusion:**

This strategy successfully engaged patients traditionally less likely to attend outpatient care, leveraging inpatient infrastructure to remove common barriers to treatment initiation. Inpatient and discharge-based HCV treatment can be a successful strategy to expand access to HCV treatment for marginalized populations. This approach, centered on multidisciplinary coordination and targeted patient engagement, demonstrates meaningful real-world feasibility. Broad implementation will require institutional investment towards dedicated personnel and processes to support identification, education, and linkage to care.

**Disclosures:**

All Authors: No reported disclosures

